# Characterizing the microbiomes of Antarctic sponges: a functional metagenomic approach

**DOI:** 10.1038/s41598-020-57464-2

**Published:** 2020-01-20

**Authors:** Mario Moreno-Pino, Antonia Cristi, James F. Gillooly, Nicole Trefault

**Affiliations:** 1 GEMA Center for Genomics, Ecology & Environment, Facultad de Ciencias, Universidad Mayor, Santiago, 8580745 Chile; 2 Department of Biology, University of Florida, Gainesville, FL 32611 USA

**Keywords:** Water microbiology, Metagenomics, Microbial ecology, Microbiome

## Abstract

Relatively little is known about the role of sponge microbiomes in the Antarctic marine environment, where sponges may dominate the benthic landscape. Specifically, we understand little about how taxonomic and functional diversity contributes to the symbiotic lifestyle and aids in nutrient cycling. Here we use functional metagenomics to investigate the community composition and metabolic potential of microbiomes from two abundant Antarctic sponges, *Leucetta antarctica* and *Myxilla* sp. Genomic and taxonomic analyses show that both sponges harbor a distinct microbial community with high fungal abundance, which differs from the surrounding seawater. Functional analyses reveal both sponge-associated microbial communities are enriched in functions related to the symbiotic lifestyle (e.g., CRISPR system, Eukaryotic-like proteins, and transposases), and in functions important for nutrient cycling. Both sponge microbiomes possessed genes necessary to perform processes important to nitrogen cycling (i.e., ammonia oxidation, nitrite oxidation, and denitrification), and carbon fixation. The latter indicates that Antarctic sponge microorganisms prefer light-independent pathways for CO_2_ fixation mediated by chemoautotrophic microorganisms. Together, these results show how the unique metabolic potential of two Antarctic sponge microbiomes help these sponge holobionts survive in these inhospitable environments, and contribute to major nutrient cycles of these ecosystems.

## Introduction

The sponge holobiont— or marine sponge-microorganisms assemblage–represents a complex symbiotic relationship that has emerged as a key model for microbe-host interactions^1^. This is due, in part, to the ancient origin of the sponge in the metazoan phylogeny^2^. Recently, metagenomic, metatranscriptomic and metaproteomic studies have provided new insights into the functional genes of sponge microorganisms, and a new understanding of the intricate metabolic connections between sponges and their microbial assemblages. From temperate and tropical systems, we have learned that bacteria in sponges are abundant and highly diverse^3^. Bacterial densities may exceed 10^9^ microbial cells per cm^3^ of sponge tissue^4^, and a single sponge species may harbor as many different taxonomic units as seawater^5^. While the species-specific assemblages found in sponges differ from the surrounding seawater^3^, like in other microbiomes, they are described by distribution curves with a few, dominant taxonomic units followed by a long tail of rare species^6,7^.

Among distantly related sponges from temperate and tropical environments, different assemblages of associated microorganisms perform similar functions^8,9^. Thus, as in other symbiosis models, the microbiomes of sponges may share a set of core functional genes rather than a common set of taxa^10–12^. These microbial assemblages display a series of molecular adaptations to the symbiotic lifestyle, establishing a functional relationship with the host. Specific molecular determinants of the host-symbiont interaction may include Eukaryotic-like protein domains (ELPs) in the form of proteins with repeated domains (e.g., ankyrin (ARP), tetratricopeptide (TPR), leucine-rich repeats (LRR)), mobile genetic elements (MGEs), and genes related to protection and the stress response (e.g., stress proteins, restriction modification (R-M), toxin-antitoxin (T-A) systems, and clustered regularly interspaced short palindromic repeats (CRISPRs))^9,13^. Together, these molecular determinants provide a suite of molecular and physiological adaptations that link the microorganisms to their sponge hosts. They may also facilitate microbial survival within the sponge, and/or mediate direct metabolic interactions^13,14^.

Further elucidating the nature of sponge holobionts is fundamental because they provide habitat for a wide range of animals, and modify the major nutrient cycles of marine ecosystems (i.e., carbon, nitrogen and phosphorus)^15–21^. Sponge holobionts couple the benthic and pelagic zones through filter feeding^22^, and as observed in coral reef ecosystems, may canalize the transfer of DOM to higher trophic levels via the existence of a “sponge-loop”^23,24^. Understanding the nature of sponge holobionts in the extreme environment of Antarctica is particularly important since they occupy up to 80% of the benthos and are considered key ecosystem-engineers^25,26^.

The first study about Antarctic sponge bacterial communities using molecular approaches by Webster *et al*. (2004) found that a significant portion of the retrieved diversity was sponge-specific, similar to other marine environments^27^. But a more recent high-throughput sequencing approach targeting Bacteria, Archaea and Eukarya, in eight Antarctic sponge holobionts (*Myxilla* sp., *Clathria* sp., *Kirkpatrickia variolosa*, *Hymeniacidon* sp., *Leucetta antarctica*, *Haliclona*, sp., *Megaciella annectens* and an undetermined Demospongiae), found the microbiomes of Antarctic sponges to be quite different from those of tropical and temperate sponges^28^. However, little is known about the functional gene repertoires of sponge microbiomes from Antarctica, their roles in the survival of the sponge holobiont, and their contributions to the cycling of major elements in Antarctic marine ecosystems. So, here we characterize the community composition and metabolic potential of microbiomes from two of the more abundant Antarctic sponges, *Myxilla* sp. and *Leucetta antarctica*, using metagenomic analyses. We aim to describe the genomic composition of these microbial assemblages and understand how this composition relates to the establishment of symbiosis, nutrient exchange, and ultimately sponge holobiont survival. To the best of our knowledge, this study represents the first functional insights of Antarctic sponge microbiomes.

## Methods

### Sample collection

Sponge samples were collected on January 2013 at two sites in Fildes Bay, King George Island, Antarctica. *Myxilla* sp. (class Demospongiae, N = 1) was sampled from site 1 (62°11′59.1″S, 58°56′35.1″W) at 5 m depth, and *L. antarctica* (class Calcarea, N = 1) was sampled from site 2 (62°11′17.7″S, 58°52′22.8″W), at 27 m depth. Sponge samples were then kept individually in plastic bags containing natural seawater at 4 °C until processing. In addition, two seawater (SW) samples were collected approximately 5 m away from the *L. antarctica* sampling location using a 5 L Niskin bottle. These samples were prefiltered on board through a 150 μm pore mesh to remove large particles, stored in an acid-washed carboy and kept in the dark until processing in the lab. Sponge and SW samples were processed within 1 hour after collection.

### Sponge and seawater treatment

Each sponge specimen was rinsed 3 times with sterilized SW, cleaned under a stereomicroscope to remove dirt and ectoparasites and stored at −80 °C until processing. From each sponge, triplicate tissue samples of ~1 cm3 were cut with a sterile scalpel blade. Separation of the microbial community intimately associated with the sponge, from that loosely attached, was conducted following the protocol described in Rodríguez-Marconi *et al*.^28^. Briefly, sponge tissue was disrupted, filtered and serially centrifuged before DNA extraction of the resulting pellet. For analyses of the surrounding planktonic community, SW samples were filtered through 20 (NY20), 3 (GSWP) and 0.2 μm (GPWP) pore size filter 47 mm in diameter (Millipore), using a Swinnex holder system and a Cole Parmer 1–600 rpm peristaltic pump. Filters were stored in 2 mL cryovials at −20 °C until DNA extraction.

### DNA extraction

Genomic DNA from the pellets obtained after sponge treatment was extracted with the PowerSoil DNA Isolation Kit (MOBIO), following the manufacturer’s instructions. For SW, 0.2 μm filters were thawed and half of them were cut into small pieces. Samples were incubated in lysis buffer (TE 1×/NaCl 0.15 M), with 10% SDS and 20 mg mL^−1^ proteinase K and incubated at 37 °C for 1 hour. DNA was extracted using 5 M NaCl and N-cetyl N, N, N trimethylammonium bromide (CTAB) extraction buffer (10% CTAB, 0.7% NaCl), incubated at 65 °C for 10 min. Protein removal was performed using a conventional phenol-chloroform method. DNA was precipitated using isopropanol at –20 °C for 1 h and resuspended in 50 μL Milli-Q water after two ethanol 70% wash steps. DNA integrity was evaluated by 0.8% agarose gel electrophoresis, quantified using a Quantifluor (Promega) with Quant-iT Picogreen (Invitrogen), and stored at −20 °C until further analysis.

### Library preparation and sequencing

Shotgun libraries were constructed using NEBNext dsDNA Fragmentase (New England Biolabs) and sequenced on the Illumina Miseq platform using a 300 cycles Miseq kit v.2 for sponge-associated microbial samples and a 500 cycles Miseq kit v.2 for the SW samples (paired-end), at the Center for Genomics and Bioinformatics, Universidad Mayor, Chile.

### Shotgun metagenomic analyses

For all metagenomes obtained, Illumina sequences were filtered based on Q > 30 quality threshold using Prinseq.^29^. Illumina adapter sequences were removed using Cutadapt^30^. Sequences containing ribosomal genes were filtered with SortmeRNA version 2.0^31^, using Silva v.119 as reference database^32^. Non-ribosomal reads were assembled using Spades assembler version 3.10^33^ with a K-mer size of 55 for *Myxilla sp*., 85 for *L. antarctica* and 33 for SW, after an evaluation of different k-mer sizes. Contigs generated from the assemblies were used to predict Open Reading Frames (ORFs) with MetaGeneMark^34^, using the default settings.

To assess the community composition of sponge microbiomes and SW communities, we performed a taxonomic annotation at read level using the Kaiju software^35^, with default settings and using the NCBI nr + euk database. This software uses protein-level classification of short high-quality reads avoiding the bias of ORF prediction. In order to compare the taxonomic composition between metagenomes, we normalized the metagenomic reads using the lowest number of reads across samples, i.e. *Myxilla sp*. (6,423,175, see Table 1 for details). For this, we randomly subsampled all other metagenomes according to this number. Confirmation of the taxonomic composition was carried out using the ribosomal reads (filtered with SortmeRNA), aligning them against the NCBI GenBank database release 209 (August 15, 2015) with DIAMOND^36^, and using the LCA algorithm implemented in MEGAN V5^37^, with the default settings.

**Table 1:** Summary of the sequencing data of the Antarctic sponge-associated microbial community and SW metagenomes.

	*Myxilla* sp.	*Leucetta antarctica*	SW
Number of high-quality reads	6,423,175	9,056,920	5,706,551
Number of contigs ≥200 bp	203,539	104,666	541,337
Estimated average coverage (%)	87	86	74
Average genome size (Mb)	6.7	6.6	2.2
N50 (>200 bp)	641	937	639
GC (%)	44.8	45.9	38.3
Number of predicted ORFs	137,177	89,044	252,171
ORFs annotated using Eggnog	24,214 (17.7%)	39,586 (44.5%)	144,564 (57.3%)
ORFs annotated using Seed	19,133 (13.9%)	33,299 (37.4%)	115,888 (45.9%)
ORFs annotated using Pfam	20,982 (15.3%)	36,579 (41.1%)	118,245 (46.9%)

To describe the metabolic potential of sponge-associated and SW communities, first, ORFs assigned as Metazoan, based on MEGAN analysis, were removed. Remaining ORFs (≥180 bp) were aligned using DIAMOND against Eggnog database v4.5^38^. Gene annotation was also computed based on the Subsystems approach with the SUPER-FOCUS tool, which uses a reduced SEED/Subsystems database^39^. In all cases, the cutoff used was E-value < 10^−5^. ORFs annotation was confirmed using Geneious version R10.2 with BLASTx against NCBI-nr database with BLOSUM62 matrix, using a Word size 3.

To evaluate the potential role of Thaumarchaeota members in nitrogen and carbon cycles, a synteny analysis of key genes for theses cycles was performed using *Nitrosopumilus* sp. close related genomes. For this, contigs containing ORFs related to ammonia oxidation and 3-Hydroxypropionate/4-Hydroxybutyrate (3HP/4HB) pathways were analyzed using Geneious version R10.2 and Trebol software (http://bioinf.udec.cl). Contigs were compared against the complete genome of “*Candidatus* Nitrosopumilus adriaticus” (accession NZ_CP011070), and “*Candidatus* Nitrosopumilus sp. AR2” (accession NC_108656) and genomic scaffolds of “*Candidatus* Nitrosopumilus Nsub” (accession NZ_LQMW00000000), “*Candidatus* Nitrosopumilus NM25” (accession NZ_BGKI00000000), “*Candidatus* Nitrosopumilus salaria” (NZ_AEXLNZ_BGKI00000000), “*Candidatus* Nitrosopumilus SJ” (accession NZ_AJVI00000000).

### Coverage and GC content

To estimate the coverage and complexity of microbial communities we use a redundancy-based approach implemented by Nonpareil^40^. Nonpareil uses the redundancy of the reads in a metagenomic dataset to estimate the average coverage and predicts the number of sequences that will be required to achieve a nearly complete coverage, which in this case was defined as ≥95%. Parameters used were a similarity threshold = 95%, minimum overlapping percentage = 50% and a maximum number of query sequences = 1000. GC content (in %) was calculated using the raw high-quality reads, filtered by >Q30, with an in-house Perl script.

### Abundance estimation, normalization and multivariate analyses

To avoid differences of gene abundance due to variation in genome size among metagenomes, a normalization by the average genome size (AGS) was performed using MicrobeCensus^41^. AGS is estimated based on the abundance of single-copy universal genes to obtain the genome equivalent across the dataset. The abundance of each annotated gene in the metagenomes was estimated based on the number of reads mapped on to predicted ORFs using Bowtie2 and SAMtools^42,43^. The abundance of each gene was normalized by its length and genome equivalent. Gene abundances are expressed in reads per kilobase per genome equivalent (RPKG). In the case of the two SW samples, RPKG values were summed, accounting for the total SW metagenome.

Exclusive and shared genes between sponge microbiomes and the SW community were computed creating a list of unique COGs using the above ORFs predicted and annotated by Eggnog database. To do so, the abundance values of each COG with the same ID were summed. Differentially abundant COG genes were calculated dividing the abundance of each gene in the sponge-associated metagenomes by the abundance of the same gene in the SW metagenome, and considering only those COG genes with a Fold Change (FC) ≥4. Additionally, analysis of orthologous groups of proteins (OGs) was performed to identify specific/unique OGs in each sponge microbiome and SW sample. To do this, all predicted proteins of the three metagenomes were merged together and proteins >50 amino acids were clustered using reciprocal BLASTp with DIAMOND and Markov Cluster algorithm (MCL) with an inflammation parameter of 1.4. OGs were annotated using Eggnog database and classified as sponge-specific only if proteins of sponge metagenomes compose the cluster. If clusters were composed only of proteins belonging to SW, they were classified as planktonic-specific proteins. Finally, shared OGs were those composed of both sponge-specific and SW proteins.

Hierarchical clustering was performed based on the abundance of each gene (RPKG values) using the Bray-Curtis dissimilarity coefficient in PRIMER v6 for Windows (PRIMER-E Ltd). Heatmap and scatterplot were generated using ggplot2 version 3.3.2 (http://ggplot2.org)^44^ under R environment version 3.3.0.

### Quantitative PCR (qPCR) estimation of gene copy numbers

The abundance of key genes related to Calvin–Benson–Bassham (CBB), Reductive Krebs (rTCA) and 3-Hydroxypropionate/4-Hydroxybutyrate (3HP/4HB) cycles, ammonia oxidation and denitrification was evaluated through qPCR. For this, degenerated primers were designed using consensus sequences obtained from the comparison of the key genes identified in *Mxyilla* sp. and *L. antarctica* metagenomes against the NCBI nr/nt database (release versions 223, 228 and 229) using BLASTn. Ten to fifty best hits retrieved were aligned using MUSCLE in the software Geneious version R10.2 using default parameters. Primers specificities, self complementary, and Tm were tested using primer-BLAST. Standards were prepared using the metagenomic DNA and cloning the products using TOPO TA Cloning Kit (Invitrogen, Life technologies). Quantitation of the standards was performed using Qubit dsDNA HS Assay Kit (Invitrogen). The qPCR was performed using KAPA SYBR FAST qPCR Kit (Kapa Biosystems, Roche) according to manufacturer’s recommendations and on a StepOne Real-Time PCR System according to conditions indicated in Supplementary Table S1. Standards were used in triplicate in a serial dilution of 1 × 10^4^ to 1 × 10^8^. Samples were run in triplicate using 1 ng μL^−1^ of DNA for *Myxilla* sp. and 0.5 ng μL^−1^ for L. *antarctica*. The data obtained were corrected according to the dilution factor of the sample and standardized according to the copy number of the housekeeping genes (*rpo*D and efl1).

### Sequence accession number

Metagenome datasets have been deposited in the NCBI Sequence Read Archive under the BioProject accession no. PRJNA528189. The Whole Genome Shotgun projects have been deposited at DDBJ/ENA/GenBank under the accessions WASW00000000 for *L. antarctica* microbiome, WASV00000000 for *Myxilla* sp. microbiome and WASX00000000 for seawater community. The versions described in this paper are versions WASW01000000 for *L. antarctica* microbiome, WASV01000000 for *Myxilla* sp. microbiome and WASX01000000 for seawater coomunity. The scritps used in the study can be accessed at 10.6084/m9.figshare.9851240.

## Results

### Antarctic sponge microbiomes differ in genomic and community composition from seawater communities

**Figure 1:**
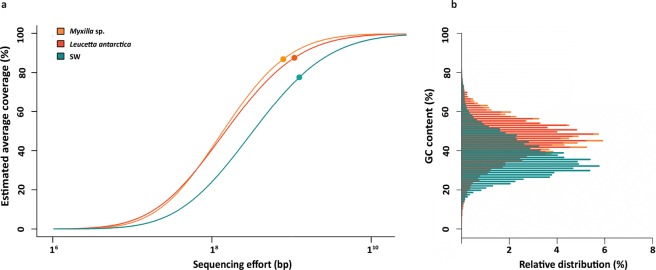
Coverage and complexity of microbiomes from the Antarctic sponges and SW communities (**a**), and their GC content (**b**). In panel a, the circles show the coverage obtained in each metagenome and the lines following the circles represent projections. In panel b, the distribution of GC content (%) for each contig assembled from the sponge microbiomes and SW metagenomes is shown.

We obtained metagenomic data from two common Antarctic sponge species, *Myxilla* sp. and *L. antarctica*, and from the surrounding SW, to compare their microbial community composition and genetic potential. Metagenomes accounted for a total of 21 Mb of sequencing data, with 15,480,095 reads for the sponge-associated metagenomes and 5,706,551 for the SW metagenomes (Table 1). Overall, microbial complexity was similar between the sponge microbiomes but both differed markedly from SW communities (Fig. 1a). This difference in complexity between the sponge and SW microbiomes was further evidenced by GC content differences of individual reads in the metagenomic datasets of sponge-associated and SW communities (Fig. 1b, Table 1).

**Figure 2:**
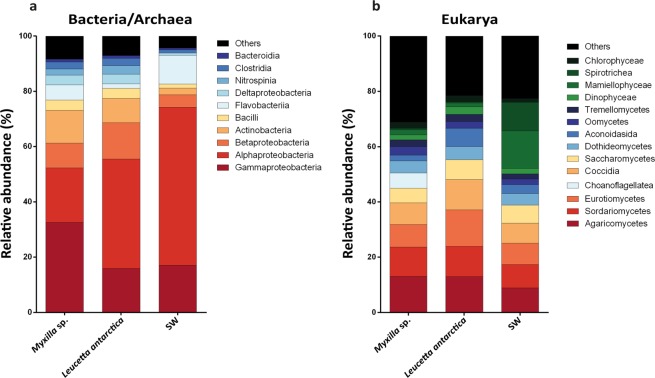
Microbial community composition of Antarctic sponges and SW based on metagenomic reads. Taxonomic composition was assigned to class level using the Lowest Common Ancestor algorithm (LCA). (**a**) Taxonomic classification for Bacteria/Archaea. (**b**) Taxonomic classification for Eukarya. All sequences assigned to Fungi were filtered.

The community composition of the sponge-associated and SW microbial communities differed notably in the Bacteria-Archaea and the Eukarya domains (Fig. 2). In the sponge-associated Bacteria-Archaea communities, the phylum Proteobacteria was the most abundant, followed by Bacteroidetes and Actinobacteria (Supplementary Table S2). At the class level, *Myxilla* sp. was mainly composed of Gammaproteobacteria (32% of total reads), Alphaproteobacteria (19%) and Actinobacteria (12%), while *L. antarctica* harbor a high abundance of Alphaproteobacteria (33%), followed by Thaumarchaeota (14%), Betaproteobacteria (13%), and Actinobacteria (3%). In contrast, SW communities were dominated mainly by Alphaproteobacteria (57%), Gammaproteobacteria (17%) and Flavobacteria (10%) (Fig. 2a). Similar taxonomic profiles were found based on ribosomal genes (Supplementary Fig. S1). The most abundant bacterial-archaeal genus associated with *Myxilla* sp. was *Cycloclasticus* (Gammaproteobacteria; Order Thiotrichales) (6% of total reads annotated at genus level), and *Nitrosopumilus* (Thaumarchaeota; Nitrosopumilales) (15%) for the *L. antarctica* microbiome. In contrast, the SW community was dominated mainly by “*Candidatus* Pelagibacter” (Alphaproteobacteria; Pelagibacterales) (34%) (Supplementary Fig. S2).

In the case of Eukarya, Fungi (Ascomycota, Basidiomycota and Fungi incertae sedis) was the most abundant group in the three metagenomes analyzed but were still significantly more abundant in the sponges (Supplementary Fig. S1). This group represented 77% and 80% of reads annotated for *Myxilla sp*. and *L. antarctica*, respectively, but only 55% for SW. Of the different fungal classes, Agaricomycetes was the most abundant, comprising on average 20% of reads among the three metagenomes (Supplementary Fig. S3a). At lower taxonomic levels, a total of 410 genera of Fungi were detected of which 307 genera (75%) were shared between the three metagenomes (Supplementary Fig. S3b). Of these, the most abundant fungal genus was *Aspergillus* (3%), followed by *Exophiala* (3%) (Supplementary Table S3). After Fungi, the most abundant eukaryotic groups detected, consisted of Oomycetes (18%), Chlorophyceae (13%), Dinophyceae (11%), and Mamiellophyceae (11%) for *Myxilla* sp., and Dinophyceae (17%), Oomycetes (15%), Chlorophyceae (14%) and Coscinodiscophyceae (12%) for *L. antarctica* (Fig. 2b, Supplementary Table S4). The most abundant eukaryotic genus associated with *Myxilla* sp. after accounting for Fungi was *Symbiodinium* (Class Dinophyceae; Order Suessiales) (6% of total reads annotated), whereas it was *Trypanosoma* (8%) in the *L. antarctica* microbiome. But for SW, eukaryotic members were dominated by Mamiellophyceae (16%) mainly composed by *Bathycoccus* (20%) (Supplementary Fig. S1). Taxonomic profiles based on ribosomal genes for the Eukarya domain were different to those based on total reads, as opposite to the Bacteria-Archaea domains (Supplementary Fig. S1).

### Antarctic sponge microbiomes display genomic adaptations to symbiotic lifestyle

**Figure 3:**
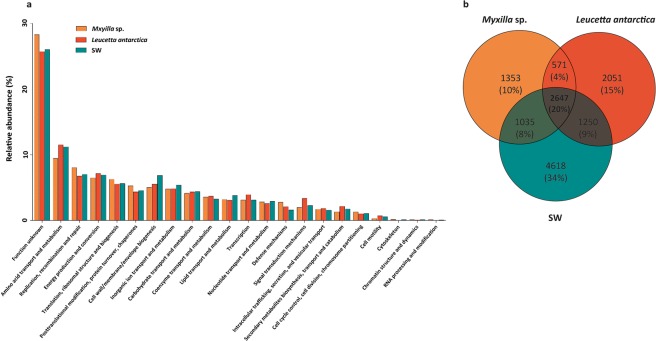
Relative abundance of Eggnog categories, and unique and share COGs between the Antarctic sponge-associated and SW communities. (**a**) Classification of proteins annotated for *Myxilla* sp., *Leucetta antarctica* microbiomes and in SW metagenome. (**b**) Uniques and shared COGs between the three metagenomes.

We annotated between 14 and 57% of the predicted ORFs (Table 1). Whereas both sponge microbiomes were composed of different assemblages of microorganisms, the relative abundance of functional genes was similar in general categories based both on EggNOG (Fig. 3a), and SEED annotation (Supplementary Fig. 4). We observed 57% functional similarity between the sponges, and 54% similarity between the sponges and the SW, based on the ORF relative abundance annotated using Eggnog. These categories include amino acid transport and metabolism, replication, recombination and repair, energy production and conversion, between others similarly abundant functions in all metagenomes.

Comparisons between sponge and SW metagenomes indicated that 29% of the total number of COGs assigned among the three metagenomes was exclusive to the sponge microbiomes. There were 1,353; 2,051 and 4,618 exclusive COGs in *Myxilla* sp., *L. antarctica* and SW, respectively, which accounted for 10%, 15% and 34% of total assigned COG’s respectively for each metagenome (Fig. 3b). Exclusive COGs represented a broad spectrum of functions, but the low RPKG values indicated that many genes occur in low abundance (Supplementary Table S5).

The most abundant sponge-specific COGs were related to symbiotic lifestyle and were functionally annotated as transposases (ENOG4110TSY, ENOG4111K9G, ENOG4111Z47), Type IV restriction endonuclease (COG2810), Type II restriction enzyme *Sfi*I (ENOG410YF7N), and Zeta toxin from the T-A system (ENOG4111XAS) (Supplementary Table S6). Further analysis of sponge-specific functions using a clustering of orthologous groups of proteins (OGs) also showed a prevalence of genomic adaptations related to a symbiotic lifestyle. The most abundant sponge-specific OGs were TPR repeat (COG0457), DNA modification methylase (COG0863), large exoprotein involved in adhesion (COG3210), and Chitinase (COG3979) -all of which represent functions related to persistence within the sponge tissue (Supplementary Table S6).

**Figure 4:**
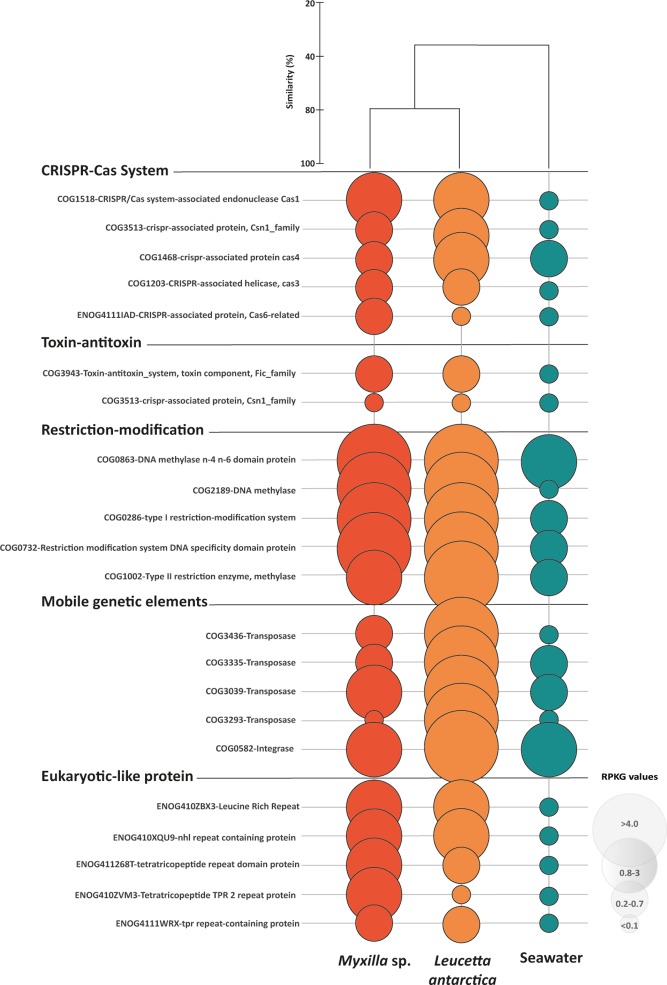
Specific functions in sponge microbiomes and SW community. Bubbles reflect abundance (RPKG) of each COG in the different functional categories. Orange bubbles represent microbiome associated with *Myxilla* sp., red bubbles *Leucetta antarctica* and green bubbles, SW. The cluster represents the percent similarity according to Bray-Curtis dissimilarity coefficient.

On the other hand, 20% of total annotated functions were COGs shared between sponge microbiomes and the SW community (Fig. 3b). Among these, 28% (751 annotated COG) and 35% (932 annotated COG) were overrepresented in *Myxilla* sp. and *L. antarctica*, respectively. This differentially abundant fraction of COGs (FC ≥ 4), were often related to the symbiotic lifestyle (Fig. 4, Supplementary Table S7). Specifically, CRISPR-associated protein, T-A and R-M systems were 73, 114 and 238 times more abundant in the sponge microbiomes than in SW. The MGEs and the ELPs (annotated as ARP, LRR and TPR repeats) were similarly over-represented in the sponge microbiomes, with up to 437 and 389 FC. In contrast, exclusive COGs in the SW community correspond to general functions, annotated as chain length determinant protein (ENOG410Y1H4); DNA-dependent RNA polymerase (COG5108); CAAX amino terminal protease family protein (COG1266); DNA polymerase (ENOG411267Q); and tryptophanase (COG3033). Accordingly, SW-specific OGs were represented by Dehydrogenase (COG1012), Dehydrogenase reductase (COG1028), Amp-dependent synthetase and ligase (COG0318), Histidine kinase (ENOG410XNMH), and Sulfatase (COG3119) (Supplementary Table S7).

### Antarctic sponge metagenomes have a broad repertoire of genes related to nutrient cycling

**Figure 5:**
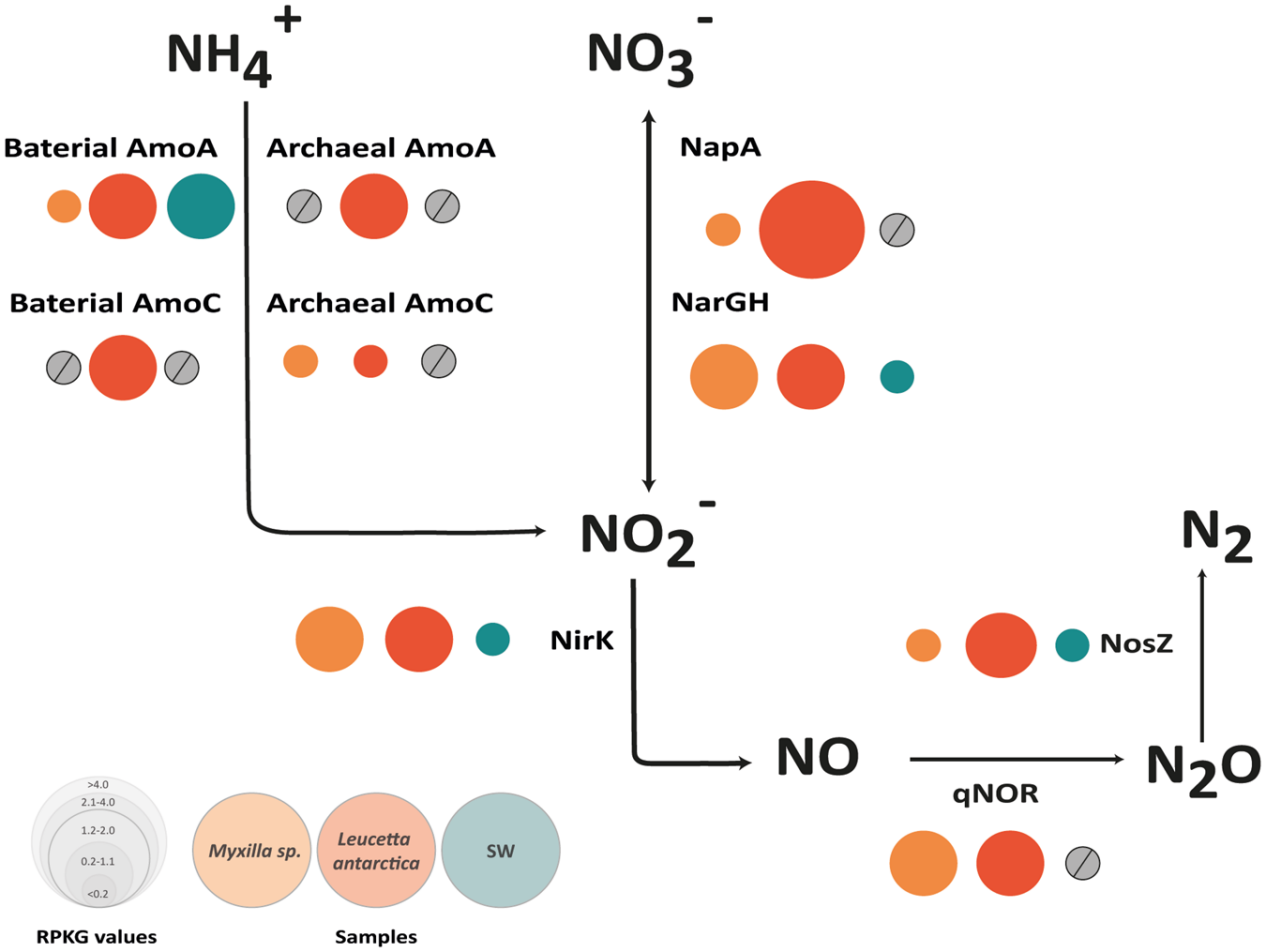
Nitrogen pathways present in Antarctic sponge symbionts and SW communities. Bubbles reflect abundance (RPKG) of each enzyme involved in nitrogen cycle normalized by the average genome size (AGS). Orange bubbles represent microbiome associated with *Myxilla* sp., red bubbles, *Leucetta antarctica* and green bubbles, SW. Grey circles represent functions that are absent in the metagenomes.

Similar to sponge microbiomes from other environments, Antarctic sponge metagenomes display a broad repertoire of genes related to nutrient cycling. In the case of the nitrogen cycle, all genes identified were more highly represented in the sponge microbiomes than in SW, i.e., ammonia oxidation, nitrite oxidation, and denitrification (Supplementary Table S8, Fig. 5). Coding genes for archaeal and bacterial ammonia monooxygenase (*amo*A and *amo*C) were enriched in the *L. antarctica* microbiome compared to *Myxilla* sp. (Fig. 5). Consistent with these results, taxonomic analysis shows that *amo* genes in *L. antarctic*a were mainly associated with *Nitrosopumilaceae* and *Rhodospirillales* members (Supplementary Table S9), and that *amo* gene clusters in this microbiome were in synteny with the available genomes of  “Candidatus Nitrosopumilus sp.” (Supplementary Fig. S8). Together, these results strongly suggest that *Nitrosopumilus* spp. are involved in the genomic potential for ammonia oxidation found in the *L. antarctica* microbiome.

Genes related to denitrification and nitrate oxidation were more abundant in the *L. antarctica* microbiome compared to *Myxilla* sp. and SW (Fig. 5). While the periplasmic nitrate reductase-encoding gene (*nap*A) and nitrous-oxide reductase-encoding gene (*nos*Z) were more abundant in *L. antarctica*, the membrane-bound respiratory nitrate reductase gene (*nar*GH), nitrate reductase (*nir*K) and nitric oxide reductase (*qnor*) were similarly abundant in both sponges. Overall, these results indicate the presence of a complete denitrification pathway in both sponge microbiomes (Fig. 5). Moreover, genes codifying for proteins involved in creatinine, creatine, and urea metabolization were also detected in both sponges. For the urea degradation, while *L. antarctica* microbiome seems to use the ATP-independent pathway, the *Myxilla* sp. microbiome apparently performs urea degradation by the ATP-dependent pathway (Supplementary Fig. 7), at least at the sampling time. qPCR results showed a similar profile compared to the metagenomic results (Supplementary Table S10).

For the sulfur cycle, oxidation pathways were detected in both sponge metagenomes (Supplementary Fig. 8), including the sulfite (reverse dissimilatory sulfate reduction *dsr*ABJKMOP, *apr*AB and *sat* genes) and sulfur oxidation (*sox*ABXYZ genes) pathways. Taxonomic analysis of *sox* genes obtained from *Myxilla* sp. microbiome reveal a high similarity (58–94%) with the genome of *Cycloclasticus* sp. (Supplementary Table S9). In the case of *L. antarctica* microbiome, *sox* genes show similarity with diverse microorganisms, including Rhizobiales, Rhodospirillales, Burkholderiales, Nitrosomonadales, Methylococcales, Gammaproteobacteria TMED119 and *Solemya elarraichensis* gill symbiont (Supplementary Table S9). Additionally, all genes for catabolization of dimethylsulphoniopropionate (DMSP) via the demethylation pathway, and transport and degradation of taurine were also detected. This indicates the potential to utilize organic sulfur compounds by both Antarctic sponge microbiomes.

For the phosphorus cycle, polymerization via the enzyme polyphosphate kinase and depolymerization of polyphosphate (polyP), using exopolyphosphatase, are also potential functions exerted by the Antarctic sponge microbiomes analysed. The enzymes polyphosphate kinase and exopolyphosphatase were enriched in both sponge microbiomes compared to SW. Furthermore, ABC phosphate transporters, codified by *pst*ABCS, were found in high abundance in the sponges analyzed here in comparison to SW (Supplementary Table S8). Further details of proteins related to biogeochemical cycling of carbon, nitrogen, sulfur and phosphorus, identified in *Myxilla* sp., *L. antarctica*, and SW are shown in Supplementary Table S8.

**Figure 6:**
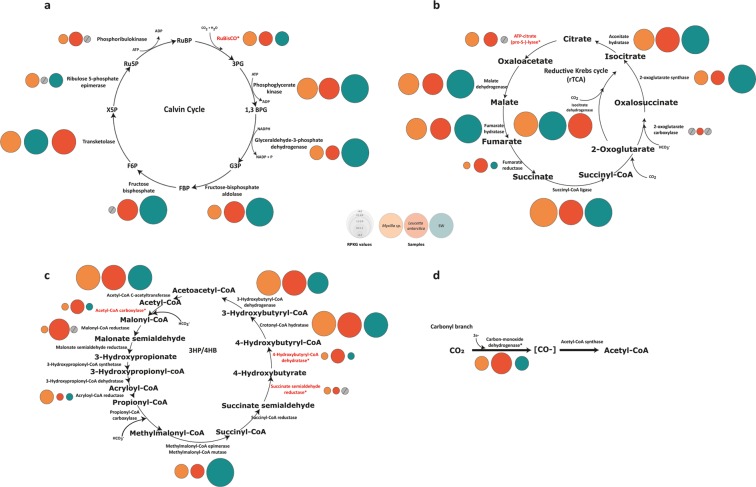
Autotrophic carbon pathways present in Antarctic sponge symbionts and SW communities. Bubbles reflect abundance (RPKG) of each enzyme involved in Calvin cycle (**a**), reductive Krebs cycle (rTCA) (**b**), 3-hydroxypropionate/4-hydroxybutyrate (3HP/4HB) cycle (**c**), and Wood–Ljungdahl pathway (**d**). Orange bubbles represent microbiome associated with *Myxilla* sp., red bubbles *Leucetta antarctica* and green bubbles, SW. Enzymes colored in red indicate key functional genes in each cycle. Grey circles represent functions that are absent in the metagenomes.

Finally, for carbon, four of the six described metabolic pathways for autotrophic carbon fixation were detected in the sponge microbiomes analyzed here: Calvin–Benson–Bassham (CBB), Reductive Krebs (rTCA), Wood–Ljungdahl (WL), and 3-Hydroxypropionate/4-Hydroxybutyrate (3HP/4HB) (Fig. 6). Genes coding for the key enzyme for the CBB cycle, Ribulose bisphosphate carboxylase (RuBisCO), were also detected in the three metagenomes, but were enriched in the *L. antarctica* microbiome (Supplementary Table S8). Taxonomic analyses of the detected RuBisCO genes revealed a dominance of bacterial form I of RuBisCO in both sponge microbiomes. All genes encoding RuBisCO recovered from the *Myxilla* sp. microbiome were assigned to the chemolithoautotroph bacteria “*Candidatus* Thioglobus sp.” (Gammaproteobacteria). In the case of *L. antarctica*, genes detected were assigned to *Nitrosospira* sp. (Betaproteobacteria), *Rhodobium* sp. (Alphaproteobacteria) and *Rhodopseudomonas* sp. (Alphaproteobacteria), and to the eukaryotic photosynthetic group of Stramenopiles and Haptophyceae (Supplementary Table S9). Abundances of key genes for the remaining three carbon fixation pathways detected in the Antarctic sponge metagenomes, i.e., rTCA, WL and 3HP/4HB, were slightly different between the sponge microbiomes, and these differences were higher for the 3HP/4HB cycle. The key enzymes for the 3HP/4HB cycle, Malonyl semialdehyde reductase, Propionyl-CoA carboxylase, and 4-Hydroxybutanoyl-CoA dehydratase, were enriched ten-fold in *L. antarctica* compared to *Myxilla* sp. (Fig. 6). Taxonomic identification of these key genes for the 3HP/4HB cycle shows between 93% and 100% identity with *Nitrosopumilus* sp. (Supplementary Table S9). Additionally, contigs containing these key genes were syntenic with the genome of *Nitrosopumilus* sp. (Supplementary Fig. S5). This is consistent with our taxonomic analysis based both on total and ribosomal reads, which indicated that *Nitrosopumilus* sp. was more abundant in *L. antarctica* than *Myxilla* sp. (see Fig. 2, Supplementary Fig. S1 and Supplementary Fig. S2). Yet in SW, only genes related to Calvin and WL cycles were found, although one of the three known genes for rTCA, and two of the three for 3HP/4HB, were also found (Fig. 6).

## Discussion

Our metagenomic analysis of the microbiomes from two ubiquitous sponge species from Antarctica, *Myxilla* sp. and *L. antarctic*a^25,45^, provide insights into sponge microbiomes from extreme environments. They are also helpful in comparing sponge holobionts from these extreme environments to temperate and tropical environments. To begin, the taxonomic composition of the two sponge microbiomes were similar to each other, and similarly different from those of surrounding SW. However, since there is no information about the individual variability of the microbiomes of the these two Antarctic sponges, comparisons with the surrounding SW could be affected by a potential effect of such variability. In general, we observed the commonly described bacterial and archaeal members of the sponge microbiota^5,46,47^. We show a dominance of Gamma- and Alphaproteobacteria, and the Archaea Thaumarchaeota, as opposed to the SW that was mainly dominated by Betaproteobacteria. Unlike most sponge-species from tropical and temperate environments, which are typically associated with Cyanobacteria, we did not detect Cyanobacterial sequences in the Antarctic sponges. The lack of Cyanobacteria in Antarctic sponges is in agreement with the absence of this taxon in marine systems from higher latitudes, and also with previous reports for Antarctic sponge microbiomes^27,28,48^. For Eukarya, the two sponge metagenomes were highly enriched in fungal sequences. Still, a large fraction of these Fungi also occurred in SW, and thus likely are non-symbiotic in nature. A similar conclusion was reached for sponges collected from the Mediterranean and North Sea^49,50^. The dominance of Fungi was unexpected since they had not been detected in our previous study^28^. Interestingly, the inferred taxonomic profiles based on total reads and on ribosomal genes fit almost exactly with expectations for Bacteria and Archaea domains based on the work of Rodríguez-Marconi *et al*.^28^, which used a different approach to analyze the same samples. But, our results significantly differed from this previous work in considering the Eukarya domain. These differences could be attributed to the lower representation of protist genomes in the database used as part of our taxonomic approach.

Additionally, the few Antarctic sponge microbiomes analysed here showed larger AGS and higher GC content in comparison to the SW planktonic community. Although genome reduction is a generalized strategy in bacterial symbionts^51^, larger genomes inside sponge bacterial communities such as those described here have been previously described^13^. This observation may be explained by the high exposition of free DNA for the sponge’s ingestion of food bacteria, which may, in turn, lead to higher levels of horizontal gene transfer (HGT) within sponges^13^. Differentiation of sponge-associated communities from surrounding SW is a common feature of sponge biology^5,9^, that reflects the intimate symbiotic relationship between the microbiome and the host invertebrate.

Functional analyses of the sponge holobionts revealed two broad categories of gene functions that differed from surrounding SW. First, sponge-associated microbiomes showed a high abundance of genes associated with the symbiotic lifestyle. These include high abundances of genes related to MGEs (e.g. transposons, phages genes), ELPs, and foreign DNA defensive mechanisms (e.g., CRISPR, T-A and R-M systems) in comparison to the planktonic microbial community. Previous work has identified these functions in genomes of bacteria associated with sponges, and in sponge metagenomes^9,52–54^. Thus, these genes could play a specific role in facilitating the symbiosis by promoting gene exchange, defending from foreign DNA, or limiting phagocytosis^55^. In doing so, they point to the possibility of genomic adaptations to the symbiotic lifestyle in *Myxilla* sp. and *L. antarctica* microbiomes, much like have been observed in sponge microbiomes from temperate and tropical environments^9,13,56^. More generally, they point to common molecular mechanisms mediating symbiosis with sponges across all environments, including Antarctica.

Second, both sponges also showed considerable functional diversity related to the cycling of sulfur, phosphorus, nitrogen and carbon. With respect to sulfur, results suggest that Antarctic sponge microbiomes analysed potentially carry out thiosulfate oxidation and sulfate oxidation, which could serve as an electron source of energy for these microorganisms^57^. Our taxonomic analysis of *sox* genes indicates that SOB in *Myxilla* sp. belong mainly to Gammaproteobacteria members, specifically to *Cycloclasticus*. This bacterial genus has been recently reported in association with the sponge *Myxilla methanophila* from hydrocarbon seeps of the Gulf of Mexico^58^. The presence of these microorganisms in two geographically distant sponges suggests that *Cycloclasticus sp*. could be a key member of the sulfur cycle, and specific to the microbiome of sponge genus *Myxilla*.

With respect to phosphorus cycling, genes coding for polyphosphate kinase, responsible for the synthesis of polyP, were detected in both Antarctic sponges. Previous work in tropical sponges suggested that symbiotic microorganisms may accumulate granules of polyP when the sponge is actively pumping, which could then function as an energy reserve for microorganisms when pumping ceases and nutrient inputs diminish^21^. Our results, though, suggest that polyP may also be synthesized for these purposes by Alpha-, Beta- and Gammaproteobacteria -abundant members of the Antarctic sponges known to possess this capacity^21^. If so, sponge holobionts may play a more prominent role than previously recognized in the phosphorus cycle of benthic Antarctic ecosystems.

Moreover, functional analyses show that Antarctic sponge microbiomes may play a significant role in nitrogen cycling by performing ammonia oxidation, nitrate oxidation, and denitrification. This is based on our detection of the archaeal and bacterial ammonia monooxygenase (*amo*A and *amo*C), the nitrate reductase-encoding gene (*nap*A), the nitrous-oxide reductase-encoding gene (*nos*Z), the membrane-bound respiratory nitrate reductase gene (*nar*GH), the nitrate reductase encoding gene (*nir*K), and the nitric oxide reductase encoding gene (*qnor*). The abundance of *Nitrosopumilus* sp. in *L. antarctica*, and the synteny of archaeal *amo* gene cluster, point to their role in ammonia oxidation. While similar results related to ammonia oxidation were not detected to *Myxilla* sp., they may still occur. Previous research in temperate and tropical sponges have similarly shown that microorganisms fulfill important roles in sponge nitrogen dynamics by metabolizing sponge waste products^9,59,60^.

Still, perhaps more than for any other nutrient, these results point to the special roles of these sponge microbiomes in carbon fixation. Unlike “phototrophic sponges” that harbor photosynthetic microorganisms to supply the sponge carbon requirements^61^, both Antarctic sponge holobionts display light-independent pathways for CO_2_ fixation mediated by chemoautotrophic microorganisms. Note that this was not the case for the SW metagenome, where the Calvin cycle was observed to be most important to carbon fixation. Only in previous work of deep-sea sponge microbiomes have chemoautotrophic microorganisms (e.g., Ammonia-Oxidizing Archaea (AOA) and Sulfur-Oxidizing Bacteria (SOB)) been described as key members^18,59,62,63^. We therefore speculate that the presence of chemoautotrophic microorganisms in the Antarctic sponges could be helpful in facing the extreme environmental conditions characterized by strong seasonal light variation. Further studies incorporating stable isotopic labeling and carbon flux measurements could help quantify the contribution of these microorganisms to carbon fixation.

## Conclusion

Our functional metagenomic analyses of two abundant Antarctic sponges, *Myxilla* sp. and *L. antarctica*, show that the microbial communities are more similar to each other, than to surrounding SW. In both cases, they show larger AGS and higher GC-content in comparison to the planktonic community. Proteobacteria and Thaumarchaeota dominated the Bacteria and Archaea domains in the sponge-associated microbial communities, whereas Fungi dominated the Eukarya. Functional analyses point to a broad range of functions associated with the symbiotic lifestyle and biogeochemical cycling. Potential functions further suggest that the microbiomes play roles in the cycling of sulfur, phosphorus, nitrogen, and carbon. In particular, these results suggest that these microbiomes utilize light-independent pathways for CO_2_ fixation that are mediated by chemoautotrophic microorganisms. The diversity of functions observed for nutrient cycling suggest that sponge microbiomes in Antarctica may play an important role in the growth and survival of sponge holobionts from these harsh environments, and more broadly, in major nutrient cycles of Antarctic marine systems.

## Supplementary information

Supplementary information.

Supplementary Tables.
