# Supplementary material for: Characterizing the microbiomes of Antarctic sponges: a functional metagenomic approach
Source: Sci Rep. 2020 Jan 20;10:645. doi: 10.1038/s41598-020-57464-2 (PMC6971038; doi:10.1038/s41598-020-57464-2)
Supplement: Supplementary file 1 — Supplementary information. [file 41598_2020_57464_MOESM1_ESM.pdf]

Manuscript

Characterizing the microbiomes of Antarctic sponges: a functional metagenomic approach.

Mario Moreno-Pino, Antonia Cristi, James Gillooly and Nicole Trefault

Supplementary information

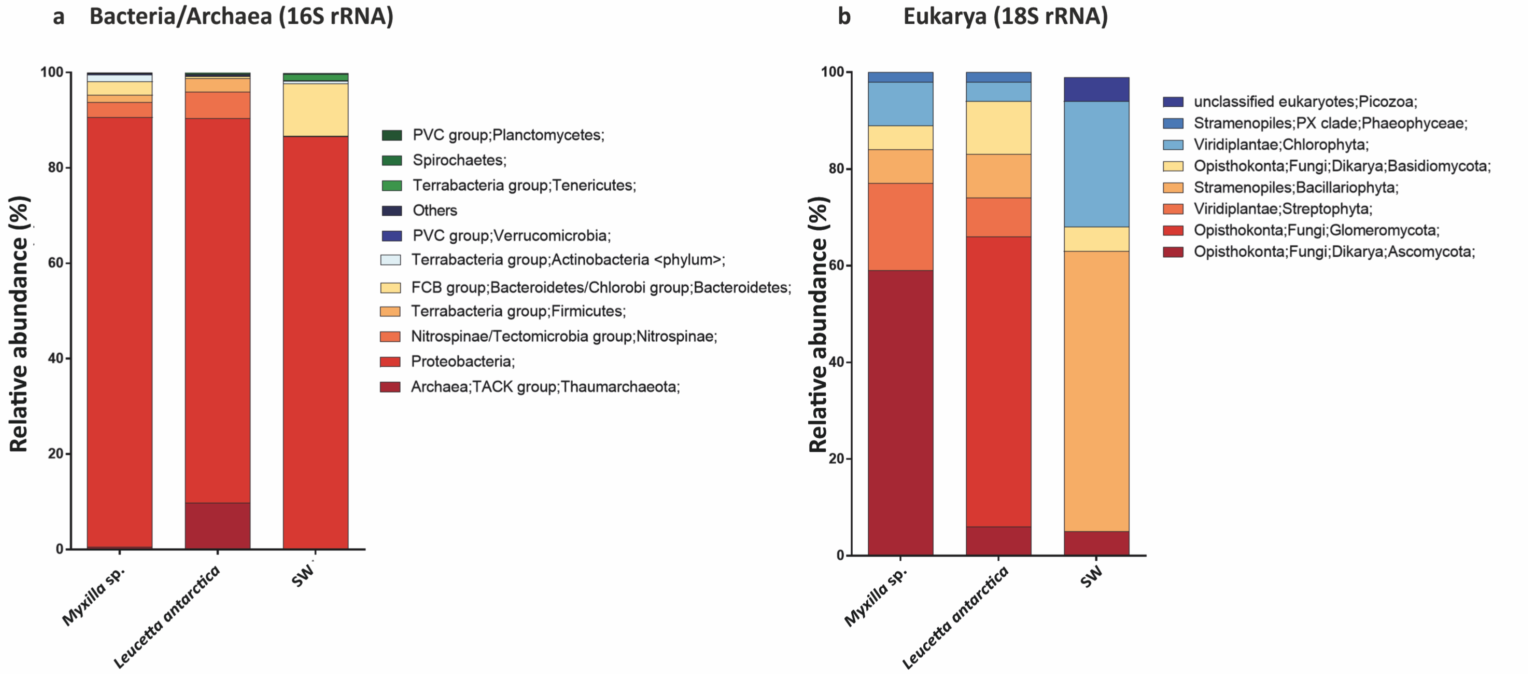

Supplementary Figure 1. Microbial composition based on ribosomal genes obtained from the metagenomes from the Antarctic sponge- associated and SW communities. Taxonomic composition was assigned to phylum level based on the Lowest Common Ancestor algorithm (LCA), implemented in MEGAN5. Taxonomic classification for Bacteria/Archaea is shown in (a) and for Eukarya domain in (b).

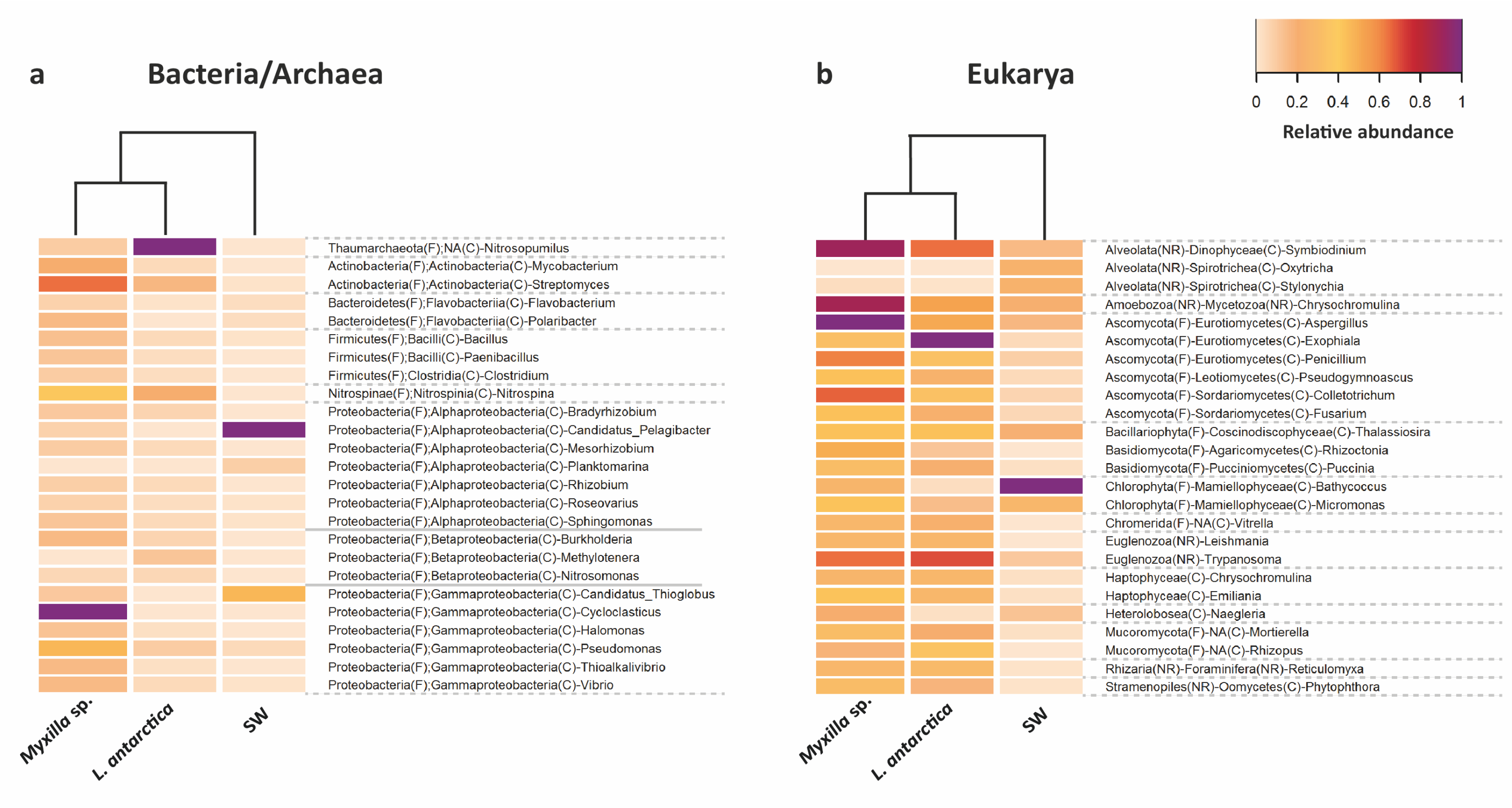

Supplementary Figure 2. Microbial composition at genus level from the Antarctic sponge- associated and SW communities. Taxonomic classification for Bacteria/Archaea is shown in (a) and for Eukarya domain in (b).

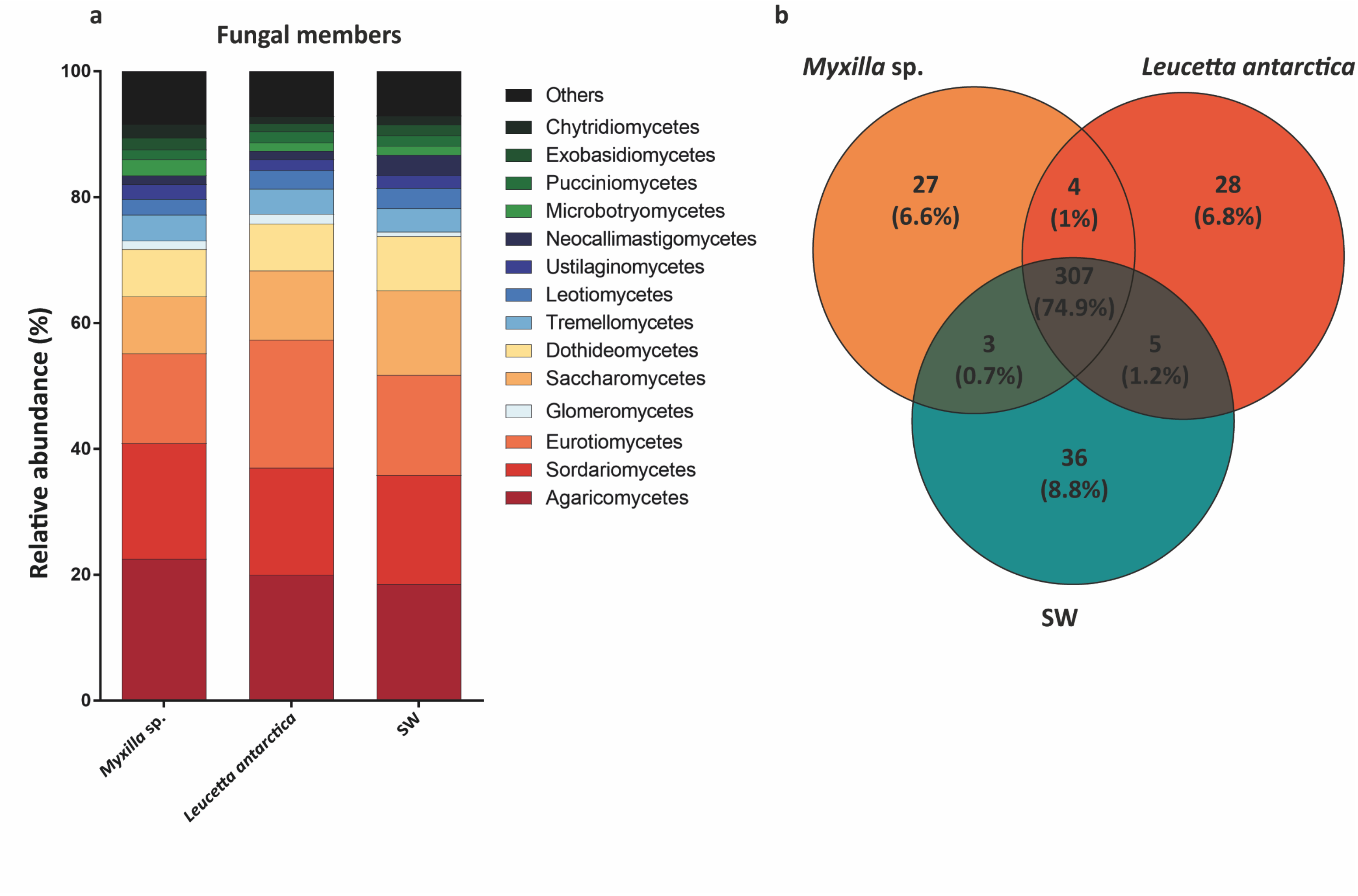

Supplementary Figure 3. Fungal microbial composition at genus level of the Antarctic sponge-associated and SW communities. (a) Relative abundance of fungal groups and (b) shared and unique fungal genera found in the metagenomes.

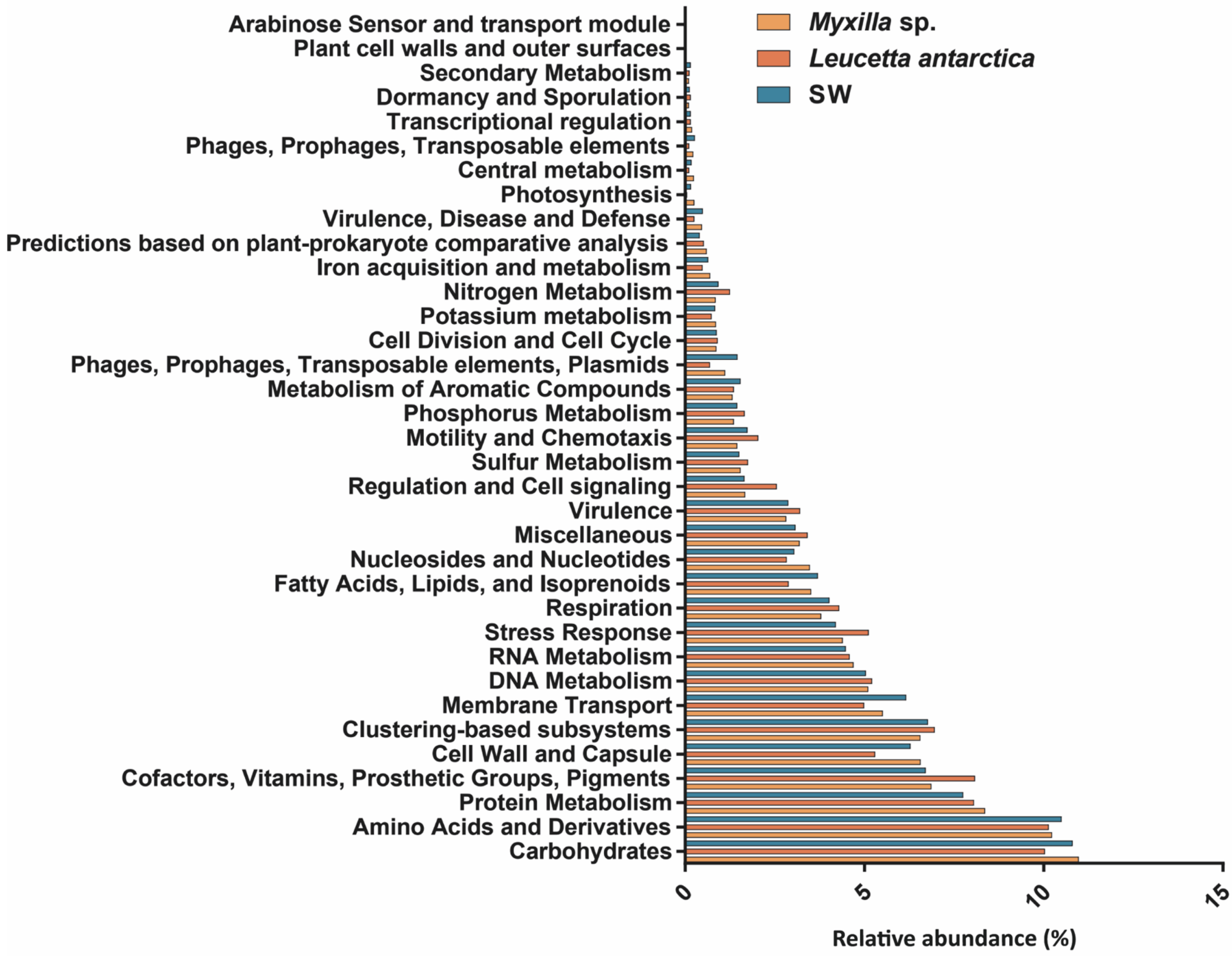

Supplementary Figure 4. Relative abundance of SEED categories between the Antarctic sponge-associated and SW communities. Classification of proteins annotated for *Myxilla* sp. and *L. antarctica* microbiomes and for the SW metagenome.

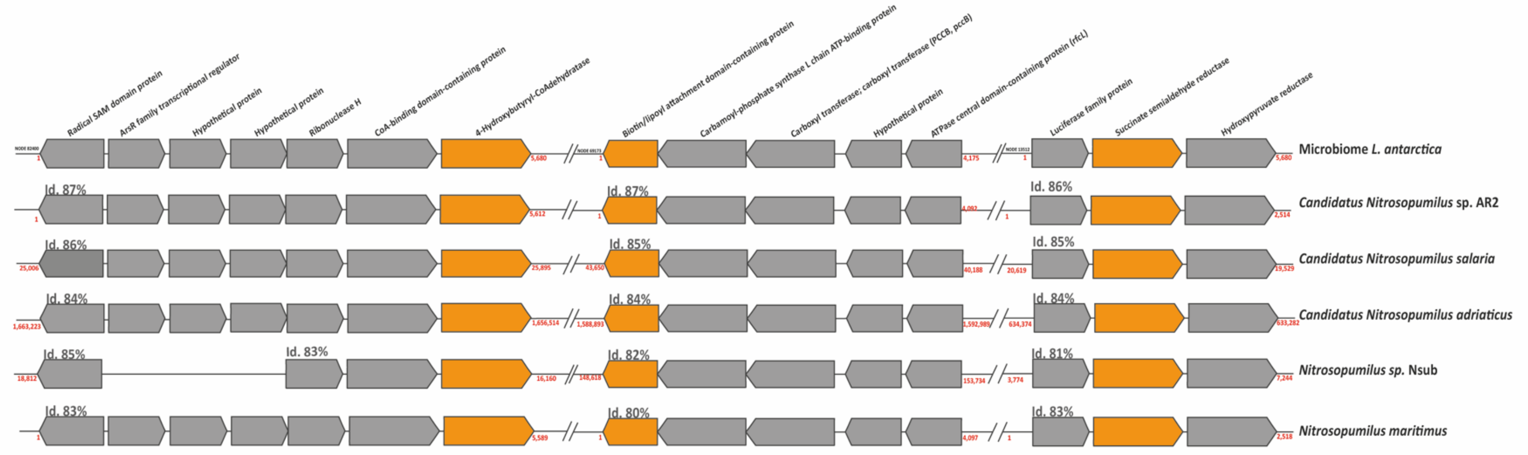

Supplementary Figure 5. Synteny analysis for the cluster of key genes related to the 3HP/4HB cycle from the *L. antarctica* microbiome. The schematic representation shows the comparison of the contigs generated from the *L. antarctica* microbiome and five genomes belonging to *Nitrosopumilus* sp. Key genes are shown in orange. Percent of identity are shown above each cluster of genes analyzed.

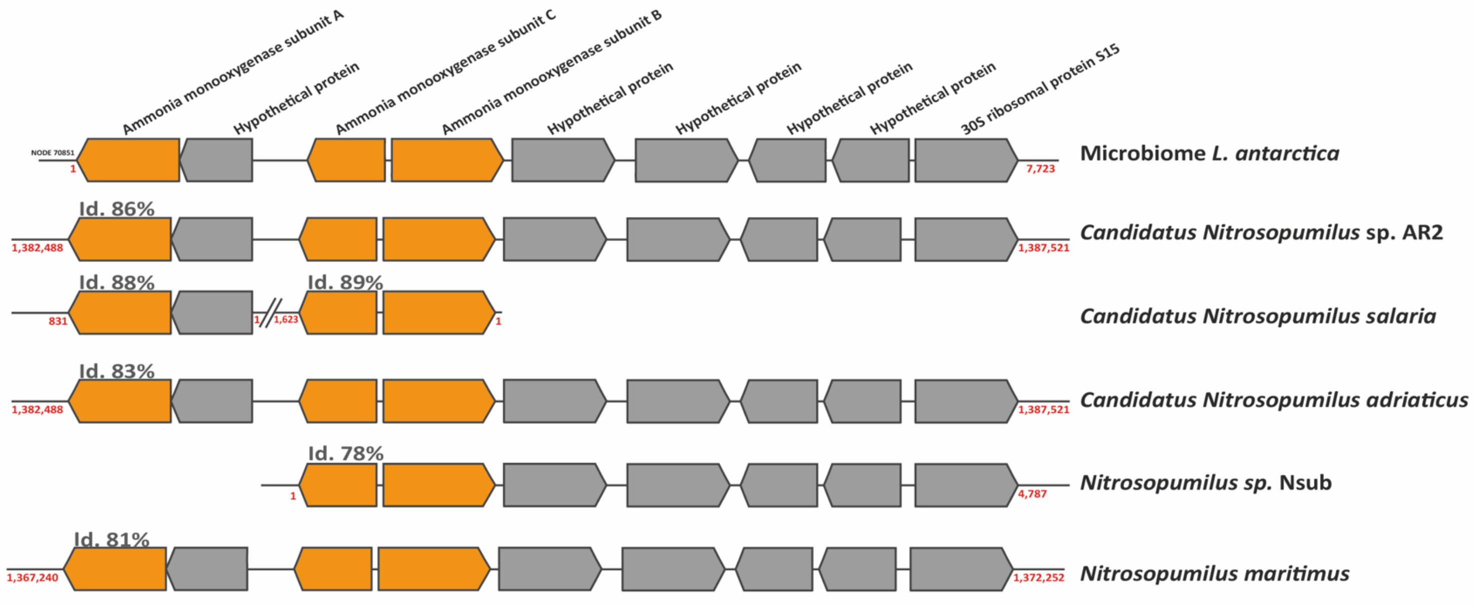

Supplementary Figure 6. Synteny analysis for the cluster of key genes related to ammonia oxidation from *L. antarctica* microbiome. The schematic representation shows the comparison of the contigs generated from the *L. antarctica* microbiome and five genomes belonging to *Nitrosopumilus* sp. Key genes are shown in orange. Percent of identity are shown above each cluster of genes analyzed.
